# The validity and reliability of a home environment preschool-age physical activity questionnaire (Pre-PAQ)

**DOI:** 10.1186/1479-5868-8-86

**Published:** 2011-08-04

**Authors:** Genevieve M Dwyer, Louise L Hardy, Jennifer K Peat, Louise A Baur

**Affiliations:** 1 Discipline of Physiotherapy, University of Sydney, Box 170 Lidcombe NSW 1825, Australia; 2 Discipline of Paediatrics and Child Health, The Children's Hospital at Westmead, University of Sydney, Locked Bag 4001 Westmead NSW 2145, Australia; 3 Prevention Research Collaboration, Level 2, K25 Medical Foundation Building, University of Sydney NSW 2006, Australia; 4 Research Consultant (Biostatistician), c/- Discipline of Paediatrics and Child Health, The Children's Hospital at Westmead, Locked Bag 4001 Westmead NSW 2145, Australia

## Abstract

**Background:**

There is a need for valid population level measures of physical activity in young children. The aim of this paper is to report the development, and the reliability and validity, of the Preschool-age Children's Physical Activity Questionnaire (Pre-PAQ) which was designed to measure activity of preschool-age children in the home environment in population studies.

**Methods:**

Pre-PAQ was completed by 103 families, and validated against accelerometry for 67 children (mean age 3.8 years, SD 0.74; males 53%). Pre-PAQ categorizes activity into five progressive levels (stationary no movement, stationary with limb or trunk movement, slow, medium, or fast-paced activity). Pre-PAQ Levels 1-2 (stationary activities) were combined for analyses. Accelerometer data were categorized for stationary, sedentary (SED), non-sedentary (non-SED), light (LPA), moderate (MPA) and vigorous (VPA) physical activity using manufacturer's advice (stationary) or the cut-points described by Sirard et al and Reilly et al. Bland-Altman methods were used to assess agreement between the questionnaire and the accelerometer measures for corresponding activity levels. Reliability of the Pre-PAQ over one week was determined using intraclass correlations (ICC) or kappa (κ) values and percentage of agreement of responses between the two questionnaire administrations.

**Results:**

Pre-PAQ had good agreement with LPA (mean difference 1.9 mins.day^-1^) and VPA (mean difference -4.8 mins.day^-1^), was adequate for stationary activity (mean difference 7.6 mins.day^-1^) and poor for sedentary activity, whether defined using the cut-points of Sirard et al (mean difference -235.4 mins.day^-1^) or Reilly et al (mean difference -208.6 mins.day^-^1) cut-points. Mean difference between the measures for total activity (i.e. Reilly's non-sedentary or Sirard's LMVPA) was 20.9 mins.day^-1 ^and 45.2 mins.day^-1^. The limits of agreement were wide for all categories. The reliability of Pre-PAQ question responses ranged from 0.31-1.00 (ICC (2, 1)) for continuous measures and 0.60-0.97 (κ) for categorical measures.

**Conclusions:**

Pre-PAQ has acceptable validity and reliability and appears promising as a population measure of activity behavior but it requires further testing on a more broadly representative population to affirm this. Pre-PAQ fills an important niche for researchers to measure activity in preschool-age children and concurrently to measure parental, family and neighborhood factors that influence these behaviors.

## Background

Physical activity is a pre-requisite for optimal growth and development in children and is also important in the prevention of chronic diseases [1-3]. In older children, physical inactivity and increasing patterns of sedentary behavior contribute to the development of overweight and obesity and its adverse health sequelae [4]. However less is known about activity behavior of very young children because there are limited tools for the measurement of physical activity and/or sedentary behavior in this age group [5-7].

No single assessment method can measure all the domains of physical activity and/or sedentary behavior [8]. Each assessment method, whether subjective or objective, has advantages and disadvantages. Questionnaires are utilized in large-scale population surveys because of relatively lower costs and participant burden [9]. There is a need for a specific questionnaire to assess activity behavior in preschool-age children [5,6]. In this age group a proxy-report tool is necessary as young children lack the cognitive capacity to assess or recall their activity [10,11]. The Preschool-age Children's Physical Activity Questionnaire (Pre-PAQ) was developed to fill this niche. Specifically it was developed to measure population estimates of activity in young children in their *home environment*. The aims of this paper are to outline the development and socioecological framework of Pre-PAQ, and to report its validity and reliability in preschool-aged children (3-5 years).

## Materials and methods

### Development of Pre-PAQ

The development of Pre-PAQ involved five strategies: (i) review of the literature; (ii) examination of existing, validated, physical activity questionnaires; (iii) consulting physical activity experts from the Australasian Child and Adolescent Obesity Research Network [12]; (iv) conducting focus groups with parents and preschool staff to assess the content and face validity of questionnaire items; and (v) pilot testing.

Pre-PAQ is a 3-day activity questionnaire designed to measure habitual physical activity and sedentary behavior in the child's home environment. Pre-PAQ has been designed under the premise that there are multidimensional influences upon young children's behavior, reflecting a socioecological framework [13-15]. The questionnaire has items related to these potential influences including: (i) parent physical activity and parenting habits and attitudes; (ii) family demographics; (iii) home and neighborhood environment; and (iv) the child's inherent activity nature (see Additional file 1: Pre-PAQ Questionnaire for complete questionnaire). A recall approach was used, in the questionnaire design, to lessen the chance that recording may alter parental activity behavior or the manner in which parents encourage their child's behavior [16].

Assessment of the child's physical activity (one week day and two weekend days) included a list of activities typical in preschool children with a response of 'Yes' or 'No' and, if 'Yes', the time the child spent in that activity. Both weekend days were included in the questionnaire as earlier parent focus groups (run as part of a different study) had indicated that activity routines at home varied more on a weekend than week days. In addition, information was attained on whether the child participated in organized activity during the week. Parents reported type of activity, duration spent in the activity and the number of times usually spent in the activity each week. Other information included how long the child spent outdoors and weather conditions on the monitored days as these are recognized influences on activity behavior [17,18].

#### Defining levels of activity

The questions related to the child's activity were classified using the Child Activity Rating Scale (CARS) [19,20] as a basis. That is, activity is classified as one of five progressive levels: completely stationary, stationary but moving a limb or the trunk, moving slowly, moving at a moderate pace, or moving quickly (see Table 1). The stationary activities of television viewing, watching DVDs, using the computer, and lying still while reading or being read to, were separated in order to identify the time spent in specific small screen recreation (SSR) activities. Time the child spent travelling in a car was also reported and included in assessment of stationary activity time (i.e. Pre-PAQ Level 1).

**Table 1:** Levels of physical activity measured by Pre-PAQ

Activity Level	Description	Type of activity
Level 1	Stationary - no movement	Sat or lay still watching TV Sat or lay still watching DVD or a video Sat or lay still (e.g. looking at books or listening to stories)
Level 2	Stationary - limb or trunk moving	Was stationary but swinging or swaying trunk (e.g. standing and singing a song) Was stationary but moving arm or leg (e.g. sitting doing puzzles or craft, digging in a sandpit or standing and kicking or throwing a ball) Played computer or electronic games
Level 3	Moving slowly	Walked at a leisurely or moderate pace Hopped, jumped, skipped or marched at an easy pace Used swing (moving self - not being pushed by another person) Rode a tricycle, bike or scooter etc. at an easy pace or slow speed Swam with support of an adult^1^
Level 4	Moving at a medium or moderate pace	Walked at a fast pace Ran or jogged slowly Rough and tumble play with moderate effort Hopped, jumped, skipped or marched at an moderate speed or effort Danced or did movement and music activities (moving around) Climbed (e.g. on play equipment, in a tree etc.) Rode a tricycle, bike or scooter etc. at an moderate pace or medium speed Swam by self (± floatation devices)^1^
Level 5	Moving at a fast pace	Walked up steep slopes Ran or jogged quickly Rough and tumble play with hard effort Hopped, jumped, skipped or marched at an fast speed or effort Rode a tricycle, bike or scooter etc. at an hard pace or fast speed

^1^Swimming activities were excluded from analyses as the child did not wear the accelerometer during this period of time.

We hypothesised that estimates of physical activity from Pre-PAQ data would demonstrate an adequate level of agreement with estimates of activity from accelerometer data, at a group summary level, and accepted that there would be differences between the two measures because the estimates were being derived from tools with different properties. As noted above, Pre-PAQ is designed as a 3-day recall questionnaire whereas accelerometer data are generally collected at 15-second to 1-minute sampling rates. Clearly, human memory cannot match this level of precision. Further, accelerometers measure incidental movement (or sedentary activity) that would not be registered as a meaningful bout of activity to an observer e.g. child moving around the home environment as part of daily routines such as walking to the bathroom, or when standing and talking with their parent. We considered an a priori adequate level of agreement to be within 30 minutes per day for sedentary level of activity, 15 minutes per day for slow-paced activity, 10 minutes per day for medium-paced activity, and 5 minutes per day for fast-paced activity, and 30 minutes per day for total activity.

### Participants

For estimating agreement between two continuously distributed variables (in this instance estimates of time measured by Pre-PAQ and accelerometry), a sample size of 100 participants gives good precision [[21], p143]. A convenience sample of 105 participant dyads (preschool-age child and their parent/guardian) were recruited via advertisements distributed to preschools statewide and within the authors' hospital and university intranet systems, and from contacts that snowballed from these strategies. Children age 3.0 to 5.9 years who had not yet commenced formal schooling were eligible to participate. Exclusion criteria were a recognized disability (physical, emotional/behavioral or intellectual) that would affect participation in physical activity and inadequate English proficiency of parents/guardians to complete the questionnaire. Informed consent was obtained from the child's parent and the study was approved by the Human Research Ethics Committees of The Children's Hospital at Westmead and The University of Sydney.

The study was conducted from December 2007 to December 2008. Prior to data collection families were visited at home or at their child's preschool. Field staff oriented the parent to the questionnaire, and demonstrated how to fit the accelerometer by using a belt and positioning the device over the child's right hip. Data collection occurred in the child's home environment corresponding to the 3-day period when the child was at home with their parent or carer.

### Reliability

To measure the test-retest reliability of the questionnaire, parents were asked to complete Pre-PAQ on two separate occasions one to two weeks apart. Reminder telephone calls, emails and/or SMS messages were used to assist with timely completion of both questionnaires.

### Criterion validity

Parents self-selected whether their child would wear an accelerometer for the period corresponding to the first administration of the questionnaire. Uni-axial MTI 7164 Actigraph motion sensors (MTI Health Services, Fort Walton Beach, FL) were used. This device has established reliability and validity in preschool-age children [22]. The devices were initialized with a 15-second sampling epoch to capture the sporadic pattern of activity in this age group [23]. Using this sampling time frame, the memory storage of the device permitted a maximum of five days data collection. Parents were asked to fit the accelerometer on their child each day during their wake time except if bathing or swimming. Children wore the accelerometer for 4-5 days with the first day's data excluded from the analyses to eliminate any reactivity to wearing the device. The variation in time wearing the accelerometer (that is, 4 or 5 days) reflected the selected weekday the child was at home with their parent, and weekend being monitored.

Accelerometer data were downloaded to a PC using the MTI Windows Actigraph software (http://www.theactigraph.com). Each file was inspected to screen the wearing pattern and ensure that the device had functioned properly. Compliance was monitored by checking for consecutive strings (20 minutes) of zero counts that were not explained by parent log of when the device had been removed (for example day time sleep or water activities) [22,24].

Only the children who wore the accelerometer for the three monitored days and who had at least six hours of recorded activity were included in the validity analyses. This approach aligns with methodological considerations advocated by Cliff et al [22]. These criteria excluded 21 children.

Time spent in activity of specific levels of intensity was estimated using cut-points described by (a) Sirard at al [25] for sedentary (SED), light physical activity (LPA), moderate physical activity (MPA) and vigorous physical activity (VPA) in 3, 4 and 5 year old children; and (b) Reilly et al [26] for sedentary and non-sedentary activity. These cut-points were selected as they had been derived specifically for preschool-age children and were based upon empirical relationships between accelerometry and direct observation (a gold standard activity measure). In order to identify stationary time, the accelerometer data were also analyzed using the cut-point 0-20 as a conservative estimate of the child being completely stationary, based upon advice of the device manufacturer. This choice of cut-point is supported by the findings of a subsequent study published by Krishnaveni et al [27]. In their study of preschool-age children they noted a range of 0-3 counts per minute for passive sitting, which would equate to a stationary activity.

Questionnaire data were entered into an Access database. Accelerometer measures were assessed for the 10-hour period between 0800 and 1800 as this time frame reflected the common wear time of the accelerometer by most participants. If the total activity reported on the questionnaire exceeded 10 hours these participants (n = 9) were removed from the criterion validity analyses.

### Statistical analysis

Data were analyzed using Statistical Package for the Social Sciences (SPSS) (Version 17 SPSS Inc., Chicago IL). MedCalc Statistical Software (Version 10.4, MedCalc Software, Mariarke, Belgium) was used for Bland-Altman tests of agreement. Tests of normality were undertaken and where data were non-normally distributed, nonparametric tests were used (kappa or Spearman's rank correlation).

#### Descriptive analyses

A three-day mean was calculated for each level of activity (minutes.day^-1^) recorded by the accelerometer, and reported by the parent. Stationary levels in the questionnaire (Pre-PAQ Levels 1-2) were summed for comparison with stationary and sedentary behavior levels from the accelerometer-derived data. Pre-PAQ stationary levels included reported time spent in the car as the accelerometer was worn during this activity. Time spent in water activities was excluded because the accelerometer was not worn at such times.

#### Reliability analyses

The reliability between the two administrations of Pre-PAQ was measured by the consistency of the item responses in the sections relating to parental report of their own and their partner's activity behavior, parenting attitudes and behaviors, pattern of car usage and active transport, facilities in the home and neighborhood environment, perceptions about the neighborhood, perceptions about the child's activity nature, reporting of the child's activity (free, unstructured activity as well as organized activity) and meal-time habits.

Reliability was assessed using intra-class correlation (ICC) for continuous variables and kappa (κ) values for categorical variables. Percent agreement of responses between the two administrations was also calculated. Interpretation of reliability was taken as < 0.20 represents poor agreement, 0.21-0.40 represents fair agreement, 0.41-0.60 represents moderate agreement, 0.61-0.80 represents good agreement and 0.81-1.00 equals very good agreement [[28], p404].

#### Validity analyses

Levels of agreement between parental reports of the child's activity time and the accelerometer (which was regarded as the 'gold standard') were analyzed as described by Bland and Altman [29]. Levels of agreement were assessed between the two measures for stationary, sedentary, light, moderate, moderate-vigorous, and light-moderate-vigorous physical (or non-sedentary) activity. Differences vs. means plots were used to assess bias between parent report of the child's activity time and accelerometer measurement.

Pearson's correlation was used to compare our findings with published validity studies, although we note the value of this statistic in estimating agreement between two measures has been questioned [29,30]. Correlations may be high but the measures may not necessarily agree and so this statistic may be misleading [29].

## Results

### Participants

**Figure 1:**
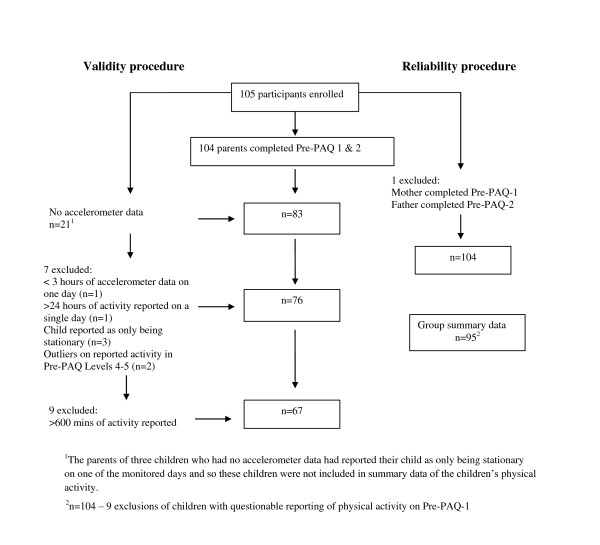
**Study design**.

Participant characteristics are shown in Table 2. The mean age of the children was 3.8 years, (SD 0.74), 87% were Caucasian and 53% were male. The parent respondent was principally the mother (92%). Of 105 families, 95% used the accelerometer. However, some children did not wear the accelerometer for the required time which resulted in different numbers of participants in the validity and reliability analyses (see Figure 1). The mean accelerometer wearing time was 9.2 hours.day^-1 ^(SD 0.79).

**Table 2:** Participant characteristics

	Reliability study (n)	Validity study (n)
**Children**	103/105 enrolled	67/105 enrolled
**Boys**	54 (52%)	35 (52%)
**Ages**		
3 year olds	37 (36%)	18 (27%)
4 year olds	46 (45%)	33 (49%)
5 year olds	20 (19%)	16 (24%)
**Parents**	103	67
**Relationship to child: mother**	95 (92%)	63 (94%)
**Socioeconomic status**^1^		
Low	7 (7%)	6 (9%)
Middle	30 (29%)	23 (34%)
High	66 (64%)	38 (57%)
**Ethnicity**		
White (Anglo-Celtic)	90 (87%)	61 (91%)
Mediterranean	6 (6%)	2 (3%)
Other ethnicity	7 (7%)	4 (6%)
**Mother's education level**		
Completed high school	10 (11%)	9 (14%)
Apprenticeship/university	85 (89%)	54 (86%)
**Marital status of parent completing Pre-PAQ**		
Married/living with partner	96 (93%)	64 (95%)
Single	7 (7%)	3 (5%)

^1^SES based upon residential postcode using the Australian Bureau of Statistics Socio-Economic Indexes for Areas (SEIFA) Index of Relative Socioeconomic Disadvantage (45) organised into tertiles.

Physical activity data from Pre-PAQ and the accelerometer (3-day mean: mins.hr^-1^) are shown in Table 3. There were no significant differences between age groups or sexes for activity levels measured by either Pre-PAQ (Age difference: F = 1.14, df = 2, 92, *P *= 0.32, Sex difference: F = 0.01, df = 1, 93, *P *= 0.92) or the accelerometer (Age difference: F = 1.02, df = 2, 73, *P *= 0.37, Sex difference: F = 0.34, df = 1, 74, *P *= 0.56) and therefore data were analyzed as one group.

**Table 3:** Activity levels measured by Pre-PAQ and accelerometry

Pre-PAQ		Accelerometer			
**Pre-PAQ level**	**3-Day mean** **(mins.hr^-1^)**	**Accelerometer categorisation**	**3-Day mean** **(mins.hr^-1^)**	**3-Day mean** **(mins.hr^-1^)** **(Reilly cut-points)**	**3-Day mean** **(mins.hr^-1^)** **(Sirard cut-points)**

Level 1-2	37.1 (34.4, 39.7)	Stationary	24.6 (CI: 23.5, 25.6)		
Level 1-2	37.1 (34.4, 39.7)	Sedentary (SED)		46.3 (CI: 45.4, 47.1)	48.9 (CI: 48.0, 49.6)
Level 3	9.7 (CI: 8.0, 11.3)	LPA			7.1 (CI: 6.6, 7.5)
Level 4	10.6 (CI: 9.3, 11.9)	MPA			2.4 (CI: 2.1, 2.7)
Level 5	2.6 (CI: 2.0, 3.4)	VPA			1.6 (CI: 1.3, 1.9)
Level 4-5	13.3 (CI: 11.6, 14.9)	MVPA			4.1 (CI: 3.6, 4.6)
Level 3-5	22.9 (CI: 20.5, 25.4)	Non-SED/LMVPA		13.7 (CI: 12.9, 14.6)	11.2 (CI: 10.3, 12.0)

#### Reliability of Pre-PAQ

The reliability of the items in the Pre-PAQ ranged from 0.31-1.00 (ICC (2, 1)) and 0.60-0.97 (κ) (Table 4). Items with lowest reliability were time the child was in the car on a weekend (Saturday: ICC (2, 1): 0.37; Sunday: ICC (2, 1): 0.31) and parental time spent in MPA on a weekend (ICC (2, 1): 0.53). Measurement error of parental activities ranged from 3.7 minutes for time spent in MPA during the week to 9.0 minutes for time spent in VPA during the weekend. Measurement error for reporting of parental screen time recreation (STR) ranged from 5.5 minutes for time spent on the computer on a weekend to 13.8 minutes for time spent watching television during the week. Parental STR activities represented time the parent spent using the computer for recreation, watching television, videos or DVDs, or playing electronic games.

**Table 4:** Reliability of Pre-PAQ

Section and item	Measurement scale	ICC (range)	Kappa (range)
**Parent**			
(1) Physical activity behaviour (Monday-Friday, Weekend)	Mins.day^-1^	0.53-0.92	
(2) Television viewing (Monday-Friday, Weekend)	Mins.day^-1^	0.70-0.88	
(3) Computer time (Monday-Friday, Weekend)	Mins.day^-1^	0.82-0.85	
(4) Parenting behaviours	9-point Likert scale	0.89-0.93	
**Family**			
(1) Car use (over a typical week)	4-point Likert scale		0.97
(2) Time child spent in car (Weekday, Saturday, Sunday)	Mins.day^-1^	0.31-0.63	
**Home and Neighborhood**			
(1) Perception of neighborhood	One of four categories		0.60-0.90
(2) Home small screen recreation items	Number of items	0.96-1.00	
**Child**			
(1) Child's activity nature	9-point Likert scale	0.87-0.93	
(2) Involvement in organised activities	Dichotomous (yes/no)		0.95
(3) Use of neighborhood facilities for activity	5-point Likert scale		0.70-0.80
(4) Pre-PAQ Levels 1-2	Mins.day^-1^	0.44	
(5) Pre-PAQ Level 3	Mins.day^-1^	0.53	
(6) Pre-PAQ Level 4	Mins.day^-1^	0.44	
(7)Pre-PAQ Level 5	Mins.day^-1^	0.64	

There was moderate to good agreement in the reporting of the child' activity with variations from an ICC (2, 1) of 0.44 (time child spent in stationary activities and time child spent in moderately-paced activities) to an ICC (2, 1) of 0.64 (time child spent in fast-paced activities). Agreement of time child spent in organized activities was very good (ICC (2, 1): 0.96-0.99) and measurement error of time child spent in organized activities ranged from 1.0-1.1 minutes. Agreement in other parental and child activities is shown in Table 4.

Items related to parenting behaviors and attitudes (ICC (2, 1): 0.89-0.93), perception of the neighborhood (κ: 0.60-0.90, % agreement: 78.0-99.1), presence of small screen recreation items in the household (ICC (2, 1): 0.96-1.0) and perception of the child's physical activity nature (ICC (2, 1): 0.87-0.93) had good to very good agreement between the two administrations of the questionnaire.

#### Validity of Pre-PAQ

Table 5 summarizes the agreement between reported activity time from the first questionnaire and the accelerometer data for the 67 children who met the inclusion criteria. Agreement was highest between Pre-PAQ Level 5 and VPA (mean difference, 1.9 mins.day^-1^) and Pre-PAQ Level 3 and LPA (mean difference, -4.8 mins.day^-1^). However the 95% limits of agreement (LoA) were wide (-37.5 to 41.3 mins.day^-1 ^and -105.5 to 96.0 mins.day^-1^, respectively).

**Table 5:** Level of agreement of time spent in the different levels of activity between Pre-PAQ and accelerometer^1^

Pre-PAQ categorisation (level)	Accelerometer categorisation	Mean difference **(mins.day^-1^)**^1^	Lower limit of agreement	Upper limit of agreement	Correlation (r)
Level 1-2	Stationary	7.6	-141.3	156.4	0.25*
Level 1-2	Sedentary (Reilly)	-208.6	-349.8	-67.5	0.28*
Level 1-2	Sedentary (Sirard)	-235.4	-383.1	-87.7	0.19
Level 3	LPA (Sirard)	-4.8	-105.4	96.0	-0.07
Level 4	MPA (Sirard)	48.2	-24.9	121.3	0.13
Level 5	VPA (Sirard)	1.9	-37.5	41.3	0.17
Level 4-5	MVPA (Sirard)	50.1	-42.9	143.1	0.17
Level 3-5	Non-sedentary (Reilly)	20.9	-121.9	163.7	0.16
Level 3-5	LMVPA (Sirard)	45.2	-103.6	194.1	0.05

^1^3-Day mean

*Significant at 0.05 level.

Level of agreement in assessing total activity (Pre-PAQ Levels 3-5 and LMVPA or non-sedentary activity) was closer when Reilly at al's cut-points were used to define the accelerometer data (mean difference 20.9 mins.day^-1^). When Sirard et al's cut-points were used the mean difference between the questionnaire and accelerometer measures was 45.2 mins.day^-1^. The mean difference between the two accelerometer categorizations (Reilly et al's non sedentary activity compared with Sirard et al's LMVPA) was 26.0 mins.day^-1^.

Agreement between Pre-PAQ Levels 1-2 and sedentary level of activity was poor whether this level was defined using Sirard et al's (mean difference -235.4 mins.day^-1^) or Reilly et al's (mean difference -208.6 mins.day^-1^) cut-points. When the categorization of accelerometer data was modified to denote stationary time (count range: 0-20), then level of agreement improved considerably (mean difference 7.6 mins.day^-1^) although the limits of agreement were still wide (95% LoA, -141.3 to 156.4 mins.day^-1^).

**Figure 2:**
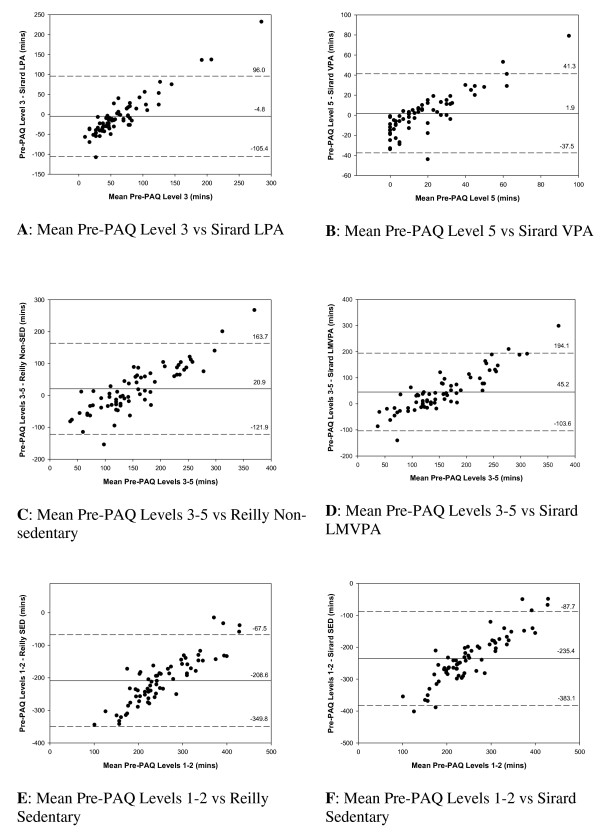
**Modified-Bland Altman plots depicting mean bias and limits of agreement between Pre-PAQ and accelerometer estimates of physical activity**.

Differences vs. mean plots of light activity (Pre-PAQ Level 3 and LPA), and moderate to fast activity (Pre-PAQ Levels 4-5 and MVPA) indicated a bias towards over-reporting by Pre-PAQ of activity time beyond certain thresholds (Figure 2). Parent report of child activity was most closely aligned with accelerometer data when the reported time on the Pre-PAQ was between 40 and 80 minutes for light activity and between 40 and 75 minutes for moderate to fast activity. The difference vs. mean plots show a systematic error in which the overestimate of activity time on the Pre-PAQ became larger as the magnitude of reported time increased. This pattern of reporting bias was also evident with fast activity (Pre-PAQ Level 5) particularly when reported Pre-PAQ Level 5 time was greater than 30 minutes.

## Discussion

Physical activity is a complex behavior and no perfect criterion measure exists [8,31]. In this study we assessed young children's activity using two assessment methods - (a) accelerometry (using two commonly accepted approaches to categorizing activity) and (b) proxy (parent) reporting on the newly developed Pre-PAQ questionnaire, in order to ascertain the validity of the latter. Pre-PAQ and accelerometry have different features in estimating the duration of physical activity levels in children. We accepted that there would be differences between the two measures because of the difference in the properties of the tools. Nonetheless, the results indicate that Pre-PAQ has moderate to very good reliability and acceptable validity detailed below.

### Reliability

Reliability coefficients on items relating specifically to the child's activity behavior, which largely represented time spent in free play or unstructured activity, ranged from moderate to good agreement for time spent in the four activity levels (Pre-PAQ Levels 1-2, 3, 4 and 5). There was very good test-retest reliability for involvement in organized activity and time spent in organized activities. This pattern of variation, with lower test-retest reliability estimates of free activity behavior compared with organized activity, has also been reported for older children [31,32]. In the older age groups, differences in reliability of activity estimates were considered acceptable because of presumed week-to-week variation in free activities, a situation that is equally applicable to young children. Thus, the test-retest differences in activity participation in this study may simply reflect real changes in activity behavior and not respondent error.

The findings of this study suggest that parent behavior was reported consistently over the two administrations of Pre-PAQ. A similar pattern of reliability in adult activity behavior was reported by Brown et al using the Active Australia Survey (AAS) in a study of middle-aged Australian women [33] and in a general adult Australian population [34]. The adult activity questions in Pre-PAQ were drawn from the AAS and the comparative results between this study and those of Brown et al suggest that the reliability of this section of Pre-PAQ is consistent with the original and modified (self-administered) versions of the AAS.

Variation in test-retest reliability was noted for reported car time. There was good agreement during weekdays but lower response consistency for car time on Saturday or Sunday. It is feasible that for most families, car use varies more on weekends than on week days, and thus the difference in reported car use may again reflect actual behavior changes.

Items relating to potential influences upon the child's activity behavior, such as parenting behaviors and attitudes, neighborhood safety and walkability, and a number of SSR items in the household, showed good to very good reliability. One would anticipate stability in these factors in the 1-2 week time frame.

### Validity

The level of agreement between Pre-PAQ and accelerometry varied between different activity levels. The measures were closest when assessing either fast-paced (mean difference: 1.9 mins.day^-1^) or slow-paced movement (mean difference: -4.8 mins.day^-1^). In assessing total activity the mean difference ranged between 20.9 mins.day^-1 ^(using Reilly's cut-points) and 45.3 mins.day^-1 ^(using Sirard's cut-points). The mean difference between the two objective measures for total activity was 26.0 mins.day^-1^. These findings suggest that Pre-PAQ has adequate validity as a population measure of physical activity. However the 95% limits of agreement were wide in each of these comparisons. Thus, while Pre-PAQ has acceptable agreement with accelerometer estimation of activity at a group level of behavior, caution should be applied in using the tool as a measure of an individual's behavior.

Pre-PAQ has better validity as a measure of physical activity rather than of sedentary behavior, as defined using the cut-points of Reilly et al [26] or Sirard et al [25]. The level of agreement between Pre-PAQ Levels 1-2 (stationary activities) and sedentary level of activity was poor. While it is well-recognized that respondents tend to under-report sedentary activities [35,36], the type of data generated using accelerometry is also a potential issue for the difference in agreement. Accelerometer data include episodes of incidental behavior (e.g. pausing for momentary conversations, toileting routines etc.). Such activities are part of every-day life and would not constitute unhealthy sedentary behavior, nor are they captured by questionnaire activity recall.

The study findings may also be influenced by the choice of accelerometry cut-points. The sedentary cut-points that we used included both low levels of activity, as well as completely stationary behavior (Sirard cut-points: 0-301 for 3 year olds, 0-363 for 4 year olds and 0-398 for 5 year olds [25], and Reilly cut-points: 0-275 for 3-5 year olds [26]). When accelerometer data were re-categorized using 0-20 counts as the cut-point for stationary activity, as opposed to sedentary activity, then the mean difference between these measures was only 7.6 mins.day^-1^. This suggests that Pre-PAQ is a valid measure of stationary activity. However, at present the cut-point for denoting stationary behavior is a theoretical construct based upon the manufacturer's advice on the Actigraph 7164 accelerometer model's sensitivity to detect movement. As noted earlier, the findings of Krishnaveni et al [27] do lend support of this theoretical cut-point. Further confirmation of the cut-point using direct observation as the comparative measure is warranted.

Pre-PAQ provides important contextual information about specific sedentary behaviors such as television viewing time, habit of eating in front of the television, and use of electronic media. These behaviors are problematic in older children and adults in terms of health outcomes compared with other light-level activities [37]. A better understanding of these specific behaviors is crucial to identify optimal habits in preschool-age children. Such important contextual information cannot be ascertained by accelerometry.

The differences vs. means plots (see Figure 2) show a systematic error in which the overestimate of activity time on the Pre-PAQ becomes larger as the magnitude of reported time increases. This pattern of bias between self-report questionnaires and accelerometry measures has also been reported in other validated self-report and proxy-report questionnaires designed for children [31,38,39]. In reporting activity (Pre-PAQ Levels 3-5), agreement with behavior measured by accelerometry is closest when the reported activity time is between 60-120 minutes. Beyond 180 minutes there is a sharp positive bias towards over-reporting of the child's activity. This finding would suggest that if respondents do report > 180 minutes of activity for their child (using Pre-PAQ) then the relationship between questionnaire data and accelerometry should be questioned.

A recent systematic review of physical activity validation studies in children aged ≤ 19 years reported low to moderate associations between the direct and indirect activity measures [40]. Correlation coefficients reported for studies using only accelerometry and questionnaires (self-report) ranged widely (from 0.03 to 0.76). In the current study, the correlation between Pre-PAQ and accelerometry was low for all levels of activity.

The results are, however, at least comparable to other proxy-report questionnaires used in a similar age group or slightly older children (see Table 6). For example, the proxy report version of the Children's Leisure Activity Study Survey, used for children aged 5-7 years, had correlations of rho = -0.06 (MPA), rho = -0.04 (VPA), and rho = -0.04 (Total Physical Activity) with accelerometry [41]. The Children's Physical Activity Questionnaire, used in children aged 4-5 years, had correlations between *r *= -0.24 and -0.10 with accelerometry using 1952 counts and 3000 counts respectively as the lower threshold for MVPA [39]. These findings suggest that Pre-PAQ is as robust as other questionnaires used in the same or slightly older age groups.

**Table 6:** Comparison of the reliability and validity of Pre-PAQ with other young children's questionnaires validated using accelerometry (Actigraph)

Tool, age group	Reliability	Validation	Correlation
CAP Questionnaire [43]: self report	Three preferred activities:	Raw movt (total counts): -	*r = *0.30
(4-9 years)	*r *= 0.41		
	Test-retest interval: 2 weeks		
CLASS [41]: proxy report	MVPA: ICC = 0.49	MPA: -	rho = -0.06
	VPA: ICC = 0.81	VPA: -	rho = -0.04
(5-6 years)	Total PA: ICC = 0.76 (frequency)	Total PA: -	rho = -0.04
		Raw movt. (counts.day^-1^): -	rho = 0.05
	Percent agreement:		
	MPA: 84.2		*NS*
	VPA: 58.6		
	Total PA: 89.2		
	Test-retest interval: 2 weeks		
CPAQ [39]: proxy report	MVPA: ICC = 0.39	Mean level of agreement	
(4-5 years)	Test-retest interval: 1 week	MVPA_1952_: -76.5 mins.wk^-1^	*r *= -0.23
		MVPA_3000 _: -235.9 mins.wk^-1^	*r *= -0.10
NPAQ [44]: proxy report	NPAQ_total _κ = 0.39	VPA _> 2818 _(mins.day^-1^): -	rho = 0.36
(Mean age in study: 5.7 years: age range not reported)	NPAQ_total _rho = 0.61	Total PA (counts.min^-1^): -	rho = 0.33
	NPAQ_total _R = 0.70		
	Test-retest interval: 2-8 weeks		
Pre-PAQ: proxy report		Mean level of agreement	
(3-5 years)	Stationary: ICC = 0.44	Stationary: 7.6 mins.day^-1^	*r = *0.25*
	Slow PA: ICC = 0.53	SED_Sirard_: -266.5 mins.day^-1^	*r *= 0.21
	Mod PA: ICC = 0.44	SED_Reilly_: -208.6 mins.day^-1^	*r = *0.28*
	Fast PA: ICC = 0.64	LPA_Sirard_: -4.8 mins.day^-1^	*r *= -0.07
	Mod-Fast PA: ICC = 0.54	MPA_Sirard_: 48.2 mins.day^-1^	*r *= 0.13
	Slow-Fast PA: ICC = 0.61	VPA_Sirard_: 1.9 mins.day^-1^	*r *= 0.17
	Test-retest interval: 1-2 weeks	MVPA_Sirard_: 50.1 mins.day^-1^	*r *= 0.17
		LMVPA_Sirard_: 45.3 mins.day^-1^	*r *= 0.05
		Non-SED_Reilly_: 20.9 mins.day^-1^	*r = *0.16
			*P < 0.05

CAP Questionnaire = Children's Activity Picture Questionnaire, CLASS = Children's Leisure Study Survey, CPAQ = Children's Physical Activity Questionnaire, Pre-PAQ = Preschool-aged Children's Physical Activity Questionnaire, NPAQ = The Netherlands Physical Activity Questionnaire for Young Children, MPA = moderate physical activity, VPA = vigorous physical activity, SED = sedentary level of activity, SED_Reilly _; sedentary level of activity using Reilly et al cut-point, SED_Sirard _; sedentary level of activity using Sirard et al cut-point, LPA = light physical activity, LPA_Sirard _; light physical activity using Sirard et al cut-point, MPA_Sirard _= moderate physical activity using Sirard et al cut-point, MVPA = moderate-vigorous physical activity, MVPA_1952 _= MVPA using 1952 counts.min^-1 ^as cut-point, MVPA_3000 _= MVPA using 3000 counts.min^-1 ^as cut-point, VPA_Sirard _= vigorous physical activity using Sirard et al cut-point, LMVPA = light-moderate-vigorous physical activity, LMVPA_Sirard _= light-moderate-vigorous physical activity using Sirard et al cut-point, Non-SED_Reilly _= non-sedentary activity using Reilly et al cut-point, rho/*r *= correlation, *P *= p-value, *NS *= not significant.

The findings of this study affirm that physical activity is a complex behavior and no perfect criterion measure exists [8,31]. Accelerometry and questionnaires both have strengths and limitations as measures of physical activity [10]. In this study, we have sought to identify how one measure relates to the other.

### Contextual information

Pre-PAQ was designed under the premise that there are multidimensional influences upon young children's behavior, reflecting a socioecological framework. This premise is supported by others [14]. It should be emphasized that Pre-PAQ has been designed to measure physical activity in the home environment as young children spend much of their time in this environment and hence are subject to the influences within this environment. Thus, Pre-PAQ also includes information about parent activity behavior, parental attitudes related to child-rearing, background culture, family structure (number, age and sex of children), and the home and neighbourhood environment, including access to and use of facilities for organised activity. The responses to questions related to culture, family structure, and home and neighborhood environment were very consistent in the test-retest assessment of Pre-PAQ (ICC (2, 1): 0.96-1.00; κ: 0.96-1.00, % agreement: 78.0-100.0). The contextual information provided by Pre-PAQ therefore should facilitate identification of factors associated with children's activity behavior.

### Limitations and modifications to Pre-PAQ

The original version of this tool included sections that assessed the child's activity preference and motor skill proficiency. The study findings showed that responses to items in these sections had very good reliability (κ = 0.70-1.00, % agreement = 80.6-100). However, the responses did not discriminate between the participants. In the section on activity preferences, parents generally reported that their child liked all the listed activities and hence this information did not assist in identifying whether activity preference influenced activity behavior. These items have been removed from the latest version of Pre-PAQ.

The motor skill proficiency items were drawn from the Ages and Stages Questionnaire, a parent-completed developmental assessment of children from birth to five years of age [42], the primary purpose of which is to identify children with developmental delay. The participants in this study were developmentally normal and consequently there was a ceiling level in this section of Pre-PAQ. The items therefore did not detect children with advanced motor skill proficiency, and hence we could not investigate the hypothesis that advanced motor skill proficiency might be associated with higher activity levels. This section has therefore also been removed from the latest version of Pre-PAQ.

In this study a convenience sample was used and the participants completed an English version of the questionnaire. We cannot assume that the findings can be generalised to non-English speaking groups. Further investigation of the usefulness of the tool with other sociocultural groups is warranted. If similar validity and repeatability are found, this would enable cross-cultural activity comparisons of young children's behavior to be undertaken. Such national and international data are essential to establish the level of young children's physical activity that is necessary to ensure optimal health.

## Conclusions

Pre-PAQ appears promising as a tool to measure activity behavior in large-scale population studies involving preschool-age children. Pre-PAQ generally has good to very good reliability. While Pre-PAQ has poor agreement with accelerometry in assessment of sedentary activity, a similar limitation has been noted for most questionnaires used to measure activity in older children and adults. On the other hand, it appears a reasonable measure of stationary activity, as a theoretical construct. Therefore it can be concluded that Pre-PAQ has adequate validity in comparison to other questionnaires used with children and youth, and appears promising as a population measure, but it requires further testing on a more broadly representative population to affirm this.

The advantages of Pre-PAQ are that it provides summary data on the nature, level and duration of a child's activity behavior. The tool also provides contextual information about potential influences on the child's activity behavior, including parental, family and neighborhood factors. This type of information is essential to identify potentially modifiable factors that can inform public health interventions to increase activity. Pre-PAQ fills an important niche for researchers to measure levels of physical activity and sedentary behavior in populations of preschool-age children and concurrently to measure parental, family and neighborhood factors that influence these behaviors.

## Competing interests

The authors declare that they have no competing interests.

## Authors' contributions

GMD designed the questionnaire, conceived and conducted the study, and drafted the manuscript. LLH assisted with the design of the questionnaire and participated in the design of the study. JKP assisted with the design of the questionnaire and the study, and advised on the statistical analysis. LAB assisted with the design of the questionnaire, and participated in the design and coordination of the study. All authors read, modified and approved the final manuscript.

## Supplementary Material

Additional file 1 **Pre-PAQ^® ^Questionnaire (as modified post assessment of its reliability and validity)**. Click here for file
