# Supplementary material for: The validity and reliability of a home environment preschool-age physical activity questionnaire (Pre-PAQ)
Source: Int J Behav Nutr Phys Act. 2011 Aug 4;8:86. doi: 10.1186/1479-5868-8-86 (PMC3162520; doi:10.1186/1479-5868-8-86)
Supplement: Additional file 1 — Pre-PAQ® Questionnaire (as modified post assessment of its reliability and validity). [file 1479-5868-8-86-S1.PDF]

the children's hospital at Westmead

The University of Sydney

Preschool-aged Children's Physical Activity Questionnaire

Pre-PAQ (Home version)

Your Name: ______________________________

Child's Name: ______________________________

Q1 What is your child's date of birth? □□-□□-□□□□

Q2 What is your child's age __________ years

Today's date □□-□□-□□□□

(day/month/year)

**Record Number:** □□□□□□ **Office Use Only**
**Office Use Only: Data Entered** □□□ **Date:** □□/□□/□□

ALL INFORMATION WILL BE KEPT STRICTLY CONFIDENTIAL

Section 1: General information

Q1 What relationship are you to the child in this study?

- ☐ Mother
- ☐ Father
- ☐ Grandparent
- ☐ Guardian
- ☐ Other (please state) ___________________

Q2 How old are you?

- ☐ <20 years
- ☐ 20-29 years
- ☐ 30-39 years
- ☐ 40-49 years
- ☐ 50-59 years
- ☐ 60-69 years
- ☐ >70 years

Q3 What is your current marital status? (please tick one box)

- ☐ Married
- ☐ Divorced
- ☐ Defacto/Living together
- ☐ Widowed
- ☐ Separated
- ☐ Never married

Q4 What is your highest level of education? (please tick *one* box)

- ☐ Completed up to Year 10 high school
- ☐ Completed Year 12 high school
- ☐ Technical or trade school certificate (TAFE)/apprenticeship
- ☐ University or tertiary qualification

Q5 What is your *partner's* highest level of education? (please tick *one* box)

- ☐ Completed up to Year 10 high school
- ☐ Completed Year 12 high school
- ☐ Technical or trade school certificate (TAFE)/apprenticeship
- ☐ University or tertiary qualification
- ☐ Not applicable

Q6 **What language(s) are spoken at home?** (please tick as many as appropriate)

☐ English ☐ Arabic

☐ Italian ☐ Vietnamese

☐ Greek ☐ Mandarin

☐ Cantonese ☐ Other (please state)

____

Q7 **What is your postcode?**____________________________

Q8 Excluding this child, **How many *other* children (siblings, step siblings, foster children etc) aged under 18 years currently live in your house?**

____

Q9 **What are their ages and gender?**

**Child's age (Years or *Months if child less than 1 year old*) – please circle**		**Gender (Male or Female)**
	Years/Months	
	Years/Months	
	Years/Months	
	Years/Months	
	Years/Months	
	Years/Months	
	Years/Months	
	Years/Months	

Section 2: Parent physical activity & parenting habits

The next questions are about any physical activities that you may have done in the last week:

	Weekdays (Monday – Friday)	Weekends (Saturday & Sunday)
**Q10a.** In the last week, how many times have you walked continuously, for at least 10 minutes (without stopping), for recreation, exercise or to get to or from places?	☐☐ times	☐☐ times
**Q10b** What do you estimate was the total time that you spent walking in this way in the last week? **Record "0" if no time spent in this activity**	☐hrs ☐☐ mins	☐hrs ☐☐ mins
**Q11a** In the last week, how many times did you do any other more moderate physical activities that you have not already mentioned? (e.g. gentle swimming, social tennis, golf etc.)	☐☐ times	☐☐ times
**Q11b** What do you estimate was the total time that you spent doing these more moderate activities in the last week? **Record "0" if no time spent in this activity**	☐hrs ☐☐ mins	☐hrs ☐☐ mins
**Q12a** In the last week, how many times did you do any vigorous physical activity which made you breathe harder or puff and pant? (e.g. jogging, cycling, aerobics, competitive tennis, gardening or heavy work around the yard etc.)	☐☐ times	☐☐ times
**Q12b** What do you estimate was the total time that you spent doing this vigorous physical activity in the last week? **Record "0" if no time spent in this activity**	☐hrs ☐☐ mins	☐hrs ☐☐ mins

These questions relate to what you did in your *FREE TIME* in THE LAST WEEK. These questions are about the time when you were SITTING and *NOT DOING CHORES*

**Q13** What do you estimate is the total time that *you* spent watching TV, videos, or DVDs as your main activity IN THE LAST WEEK? Please do not include time when the TV was switched on and you were doing something else such as preparing a meal	☐hrs ☐☐ mins	☐hrs ☐☐ mins
**Q14** What do you estimate is the total time that *you* spent playing electronic games IN THE LAST WEEK? Please circle which electronic games were used<br>PlayStation, Nintendo, XBOX, Wii II	☐hrs ☐☐ mins	☐hrs ☐☐ mins
**Q15** What do you estimate is the total time that *you* spent using the computer at home *in your free time* IN THE LAST WEEK? (NOT including use for work)	☐hrs ☐☐ mins	☐hrs ☐☐ mins

Q16 **How much do you agree with the following statements?**
Please tick *one* box for each statement

	Never	Rarely	Occasionally	Frequently	All the time
I encourage my child to play outside when the weather is suitable	☐	☐	☐	☐	☐
I am physically active with or in front of my child	☐	☐	☐	☐	☐
I limit what my child does as I worry that he/she may injury themselves	☐	☐	☐	☐	☐
I focus upon my child developing their basic learning skills such as numbers and letters.	☐	☐	☐	☐	☐
My work schedule or other commitments limit the time I have to play with my child.	☐	☐	☐	☐	☐

Section 3: Home and neighbourhood

Q17 **What best describes your backyard?** (please tick *one* response)

☐ No yard at all

☐ No private yard

☐ A small yard

☐ A medium yard (eg. a standard block of land)

☐ A large yard (eg. ¼ acre/1000m^2 or more)

Q18 **Do you have access to any of the following facilities within your backyard or home environment? (**please tick as many responses as apply)

	Yes	No
Play equipment (e.g. swing set, slide, climbing gym)	☐	☐
Pool or spa	☐	☐
Area suitable to ride a tricycle, bike or scooter etc.	☐	☐

Q19 How many of the following items are in your home?

	How many?
Television sets	☐
DVD or video players	☐
Electronic games (e.g.Play Station, Nintendo, X-Box, Wii II)	☐
Computers (laptop or desktop)	☐

Do you have the following connections in your home

	Yes	No
Internet	☐	☐
Pay television (eg. Foxtel)	☐	☐
**Q20 Is there a television in your child's bedroom?**	☐	☐

Q21 Does your local neighbourhood have the following places or facilities where your child can be play and be physically active? (please tick as many responses as apply)

	Yes	No	Not sure
Open areas such as beaches, rivers, natural reserves	☐	☐	☐
Public park or oval	☐	☐	☐
Playground	☐	☐	☐
Public swimming pool	☐	☐	☐
Gym that offers programs for young children e.g. kindergym, playgym etc.	☐	☐	☐
Club that offers activities/sports for young children e.g. soccer, dance etc.	☐	☐	☐

Q22 How much do you agree with the following statements?
(please tick one response for each statement)

	Strongly Agree	Agree	Disagree	Strongly disagree
It is safe for my child to play outdoors in my neighborhood (if supervised).	☐	☐	☐	☐
There are usable footpaths on most of the streets in my local area.	☐	☐	☐	☐
There are major barriers or dangers to walking with my child in my neighborhood that make it hard to get from place to place (for example, major roads, railway lines, canals, storm water drains or rivers).	☐	☐	☐	☐
There is so much traffic along the streets that it makes it difficult or dangerous to walk with my child in my neighborhood.	☐	☐	☐	☐
There are sufficient traffic lights or pedestrian crossings to make it safe to walk with my child around my neighborhood.	☐	☐	☐	☐
The level of crime in my neighborhood makes it unsafe to go on walks with my child during the day.	☐	☐	☐	☐
The local shop(s) are within easy walking distance of my home.	☐	☐	☐	☐
There are dangers (e.g. dogs, undesirable people) in the local park(s) so I avoid taking my child there.	☐	☐	☐	☐

The following questions are about how you and your family get around your neighbourhood

Q23 How long did your child spend in a car, in total, LAST WEEK (weekdays + Saturday + Sunday)?

**Weekdays (Monday-Friday)**	☐ hrs ☐☐ mins
**Saturday**	☐ hrs ☐☐ mins
**Sunday**	☐ hrs ☐☐ mins

Q24 How often did your child walk (e.g. to friends, shops, park, child care etc) to get around your neighbourhood LAST WEEK? (please tick *one* box)

☐ Not at all ☐ 1-2 days ☐ 3-4 days ☐ 5-7 days

Section 4: Your child

Q25 Which child care facilities or services did your child attend LAST week?

None ☐ ⇨ **Please proceed to Q26**

	Yes	No	Number of days attending (include ½ days)	Amount of time (total hours)
Informal child care (for example, grandparents, friends, nanny)	☐	☐	________	________
Family Day Care	☐	☐	________	________
Long Day Care	☐	☐	________	________
Occasional Care	☐	☐	________	________
Preschool	☐	☐	________	________

Q26 Does your child have any physical or medical condition that *affects* his/her ability to play and be physically active?

☐ No

☐ Yes (Please state nature of condition)________________________

Q27 How well do these statements describe your child?
(Please tick one box and one response for each statement)

	Never	Rarely	Occasionally	Frequently	All the time
My child has a very active nature	☐	☐	☐	☐	☐
My child needs me to motivate him/her to play	☐	☐	☐	☐	☐
My child needs company (e.g., friends, siblings, parents, adults) to be motivated to play	☐	☐	☐	☐	☐

Keep going you are half way there!!

Q28 How active would you rank your child to be compared with other children your child's age?

☐	☐	☐	☐	☐
A lot less active	Less active	Same	More active	A lot more active

Q29 Does your child eat his/her meals in front of the television?

☐	☐	☐	☐
Not at all or rarely	1 meal a day	2 meals a day	3 meals a day

Q30 Does your child attend any organised PHYSICAL ACTIVITY (e.g. swimming, gym, dance) during the week?

☐ Yes ☐ No

If yes, how many hours does your child spend in these activities during the week?

Name of organised activity	Total time usually spent in that activity each week
Swimming	☐hrs ☐☐ mins
Gym-type program (e.g. kindagym)	☐hrs ☐☐ mins
Dance/Physical culture	☐hrs ☐☐ mins
Sport<br>Name of sport: ____________________	☐hrs ☐☐ mins
Other:<br>Name of activity: ____________________	☐hrs ☐☐ mins

Q31 How often does your child use the facilities listed below to play and be physically activity, in a typical month, when the weather is suitable?
(please tick as many responses as apply)

	Daily	A few times a week	Once a week	A few times a month	Once a month	Rarely
Open areas such as beaches, rivers, natural reserves	☐	☐	☐	☐	☐	☐
Park or oval	☐	☐	☐	☐	☐	☐
Public playground	☐	☐	☐	☐	☐	☐
Swimming pool (public or private)	☐	☐	☐	☐	☐	☐

Think about where your child spent his/her time *YESTERDAY*.

Note: If yesterday was a Saturday or Sunday, or a day when your child was in formal care then this question refers to the *most recent* WEEK DAY (i.e. Monday-Friday) *when your child was at home with you*

Q32 What was the weather like YESTERDAY? (please tick *one* response)

Fine to play outdoors ☐

Too wet to play outdoors ☐

Too hot or humid to play outdoors ☐

Too cold to play outdoors ☐

Q33 How much time did your child spend outdoors in active play YESTERDAY?
(record "0" if your child did not spend time playing outside)

☐ hours ☐☐ mins

Q34 Which of the following activities did your child do YESTERDAY?
(record "0" for any activities that your child did not do)

	**Did your child do this activity?**		**Total time spent in activity**
	**Yes**	**No**	***Hours/Minutes***
Sat or lay still watching TV	☐	☐	☐hrs ☐☐ mins
Sat or lay still watching a DVD or a video	☐	☐	☐hrs ☐☐ mins
Sat or lay still (e.g. looking at books or listening to stories)	☐	☐	☐hrs ☐☐ mins
Played computer or electronic games<br><br>Please circle which electronic games were used:<br>PlayStation, Nintendo, Gameboy, XBOX<br>i-Pad, Wii II, i-toy, Other	☐	☐	☐hrs ☐☐ mins
Was **stationary** but moving a part of the body such as swinging or swaying trunk (e.g. standing and swaying to a song) or moving arm or leg (e.g. sitting doing puzzles or craft, digging in a sandpit or standing and kicking or throwing a ball, doing movements to a song)	☐	☐	☐hrs ☐☐ mins
Walked at a **leisurely or moderate pace** (for any reason – not just when going on a walk)	☐	☐	☐hrs ☐☐ mins

	Did your child do this activity?		Total time spent in activity
	**Yes**	**No**	***Hours/Minutes***
Walked at a **fast pace**	☐	☐	☐hrs ☐☐ mins
Walked up steep slopes	☐	☐	☐hrs ☐☐ mins
Ran or jogged **slowly**	☐	☐	☐hrs ☐☐ mins
Ran or jogged **quickly**	☐	☐	☐hrs ☐☐ mins
Rough & tumble play with **moderate effort**	☐	☐	☐hrs ☐☐ mins
Rough & tumble play with **hard effort**	☐	☐	☐hrs ☐☐ mins
Hopped, jumped, skipped or marched at an **easy pace**	☐	☐	☐hrs ☐☐ mins
Hopped, jumped, skipped or marched with **moderate speed or effort**	☐	☐	☐hrs ☐☐ mins
Hopped, jumped, skipped or marched with **fast speed or hard effort**	☐	☐	☐hrs ☐☐ mins
Danced or did movement and music activities (moving around)	☐	☐	☐hrs ☐☐ mins
Climbed (e.g. on play equipment, in a tree etc.)	☐	☐	☐hrs ☐☐ mins
Used swing (moving self. *Not* being pushed by another person)	☐	☐	☐hrs ☐☐ mins
Rode a tricycle, bike or scooter etc. at an **easy pace or slow speed**	☐	☐	☐hrs ☐☐ mins
Rode a tricycle, bike or scooter etc.at an **moderate pace or medium speed**	☐	☐	☐hrs ☐☐ mins
Rode a tricycle, bike or scooter etc.at a **hard pace or fast speed**	☐	☐	☐hrs ☐☐ mins
Swam by self (± floatation devices)	☐	☐	☐hrs ☐☐ mins
Swam with support of an adult	☐	☐	☐hrs ☐☐ mins
Other (please state)	☐	☐	☐hrs ☐☐ mins
Other (please state)	☐	☐	☐hrs ☐☐ mins

Think about where your child spent his/her time LAST WEEKEND (Saturday-Sunday)

Q35 What was the weather like LAST WEEKEND?
(please tick *one* response)

	SATURDAY	SUNDAY
Fine to play outdoors	☐	☐
Too wet to play outdoors	☐	☐
Too hot or humid to play outdoors	☐	☐
Too cold to play outdoors	☐	☐

Q36 How much time did your child spend outdoors in active play LAST WEEKEND?
(record "0" if your child did not spend time playing outside)

SATURDAY	SUNDAY
☐ hours ☐☐ mins	☐ hours ☐☐ mins

Q37 Which of the following activities did your child do LAST WEEKEND?
(record "0" for any activities that your child did not do)

	Saturday			Sunday		
	**Did your child do this activity?**		**Total time spent in activity**	**Did your child do this activity?**		**Total time spent in activity**
	**Yes**	**No**	***Hours/Minutes***	**Yes**	**No**	***Hours/Minutes***
Sat or lay still watching TV	☐	☐	☐hrs ☐☐ mins	☐	☐	☐hrs ☐☐ mins
Sat or lay still watching a DVD or a video	☐	☐	☐hrs ☐☐ mins	☐	☐	☐hrs ☐☐ mins
Sat or lay still (e.g. looking at books or listening to stories)	☐	☐	☐hrs ☐☐ mins	☐	☐	☐hrs ☐☐ mins
Played computer or electronic games<br><br>Please circle which electronic games were used:<br>PlayStation, Nintendo, Gameboy, XBOX, i-PAD<br>Wii II, i-toy, Other	☐	☐	☐hrs ☐☐ mins	☐	☐	☐hrs ☐☐ mins
Was **stationary** but moving a part of the body such as swinging or swaying trunk (e.g. standing and swaying to a song) or moving arm or leg (e.g. sitting doing puzzles or craft, digging in a sandpit or standing and kicking or throwing a ball, doing movements to a song)	☐	☐	☐hrs ☐☐ mins	☐	☐	☐hrs ☐☐ mins
Walked at a **leisurely or moderate pace.** (for any reason – not just when going on a walk)	☐	☐	☐hrs ☐☐ mins	☐	☐	☐hrs ☐☐ mins

	Saturday			Sunday		
	**Did your child do this activity?**		**Total time spent in activity**	**Did your child do this activity?**		**Total time spent in activity**
	**Yes**	**No**	***Hours/Minutes***	**Yes**	**No**	***Hours/Minutes***
Walked at a **fast pace**	☐	☐	☐hrs ☐☐ mins	☐	☐	☐hrs ☐☐ mins
Walked up steep slopes	☐	☐	☐hrs ☐☐ mins	☐	☐	☐hrs ☐☐ mins
Ran or jogged **slowly**	☐	☐	☐hrs ☐☐ mins	☐	☐	☐hrs ☐☐ mins
Ran or jogged **quickly**	☐	☐	☐hrs ☐☐ mins	☐	☐	☐hrs ☐☐ mins
Rough & tumble play with **moderate effort**	☐	☐	☐hrs ☐☐ mins	☐	☐	☐hrs ☐☐ mins
Rough & tumble play with **hard effort**	☐	☐	☐hrs ☐☐ mins	☐	☐	☐hrs ☐☐ mins
Hopped, jumped, skipped or marched at an **easy pace**	☐	☐	☐hrs ☐☐ mins	☐	☐	☐hrs ☐☐ mins
Hopped, jumped, skipped or marched with **moderate speed or effort**	☐	☐	☐hrs ☐☐ mins	☐	☐	☐hrs ☐☐ mins
Hopped, jumped, skipped or marched with **fast speed or hard effort**	☐	☐	☐hrs ☐☐ mins	☐	☐	☐hrs ☐☐ mins
Danced or did movement and music activities (moving around)	☐	☐	☐hrs ☐☐ mins	☐	☐	☐hrs ☐☐ mins
Climbed (e.g. on play equipment, in a tree etc.)	☐	☐	☐hrs ☐☐ mins	☐	☐	☐hrs ☐☐ mins
Used swing (moving self. *Not* being pushed by another person)	☐	☐	☐hrs ☐☐ mins	☐	☐	☐hrs ☐☐ mins

			Saturday			Sunday
	**Did your child do this activity?**		**Total time spent in activity**	**Did your child do this activity?**		**Total time spent in activity**
	**Yes**	**No**	***Hours/Minutes***	**Yes**	**No**	***Hours/Minutes***
Rode a tricycle, bike or scooter etc. at an **easy pace or slow speed**	☐	☐	☐hrs ☐☐ mins	☐	☐	☐hrs ☐☐ mins
Rode a tricycle, bike or scooter etc. at an **moderate pace or medium speed**	☐	☐	☐hrs ☐☐ mins	☐	☐	☐hrs ☐☐ mins
Rode a tricycle, bike or scooter etc. at a **hard pace or fast speed**	☐	☐	☐hrs ☐☐ mins	☐	☐	☐hrs ☐☐ mins
Swam by self (± floatation devices)	☐	☐	☐hrs ☐☐ mins	☐	☐	☐hrs ☐☐ mins
Swam with support of an adult	☐	☐	☐hrs ☐☐ mins	☐	☐	☐hrs ☐☐ mins
Other (please state)	☐	☐	☐hrs ☐☐ mins	☐	☐	☐hrs ☐☐ mins
Other (please state)	☐	☐	☐hrs ☐☐ mins	☐	☐	☐hrs ☐☐ mins

Thank you for completing this questionnaire
